# *Streptococcus dysgalactiae* as a cause of peripartum infections - a population-based cohort study with phylogenetic analysis of hospital clusters

**DOI:** 10.1007/s10096-025-05284-5

**Published:** 2025-10-04

**Authors:** Matilda Dooley, Erik Senneby, Omar Sigurvin Gunnarsson, Anja Carblom, Ann-Cathrine Petersson, Magnus Rasmussen

**Affiliations:** 1 Department of Infectious Diseases, Skåne University Hospital, Lund, Sweden; 2 Clinical Microbiology, Department of Translational Medicine, Faculty of Medicine, Lund University, Malmö, Sweden; 3 Department of Clinical Microbiology, Infection Control and Prevention, Region Skåne, Lund, Sweden; 4 Department of Obstetrics and Gynecology, Skåne University Hospital, Lund and Malmö, Sweden; 5 Perinatal and Cardiovascular Epidemiology, Clinical Sciences Malmö, Lund University Diabetes Centre, Lund University, Malmö, Sweden; 6 Department of Clinical Sciences Lund, Division of Infection medicine, Lund University, Lund, Sweden

**Keywords:** Streptococcus dysgalactiae, Peripartum infections, *Emm-*types, Hospital cluster, Whole-genome sequencing

## Abstract

**Purpose:**

We aimed to investigate the incidence and clinical features of *Streptococcus dysgalactiae* peripartum infections (SDPI), and to investigate the distribution of *emm-*types in relation to disease severity and the genetic relatedness of isolates from hospital clusters.

**Materials and methods:**

Patients with growth of *S. dysgalactiae* in a genital or wound culture, collected between January 2014 and September 2020 at departments for gynecology and obstetrics, were identified. For inclusion, patients had to be pregnant, or given birth, or undergone an abortion within 42 days prior to debut of symptoms. All isolates had previously been *emm-*typed. A cluster was defined as two or more patients with *S. dysgalactiae* of the same *emm-*type admitted to the same hospital within a 30-day period. The cluster isolates were subjected to whole genome sequencing (WGS).

**Results:**

The final study cohort comprised 130 patients. The incidence of *S. dysgalactiae* postpartum infection was approximately 1 case/1000 births. The patients fulfilled criteria for endometritis (*n* = 94), postpartum fever (*n* = 15), wound infection (*n* = 8) or chorioamnionitis (*n* = 4). Most patients with endometritis (87%) had onset of symptoms > 48 h post-partum. The most common *emm-*type was *stG62647* (*n* = 41). Thirteen hospital clusters were identified, of which only three had bacterial isolates that were closely genetically related (0–6 SNP).

**Conclusion:**

Our findings demonstrate that SDPI impact a relatively large number of patients. No patient was critically ill, but the morbidity appeared to be substantial. Hospital outbreaks of SDPI are rare, but when suspected, WGS should be employed to investigate relatedness between isolates.

## Introduction

*Streptococcus dysgalactiae* is a β-haemolytic *Streptococcus* belonging to Lancefield group C or G, colonizing the skin, oropharynx, gastrointestinal and female genital tract. *S. dysgalactiae* has in the past decades come to be known as an important human pathogen and causes infections with a similar clinical presentation as *Streptococcus pyogenes*, including skin and soft tissue infection, necrotizing fasciitis and streptococcal toxic shock-like syndrome [1, 2].

Maternal peripartum infections encompass endometritis, intraamniotic infection, wound infections, and maternal sepsis. Endometrial cultures from patients with endometritis and chorioamnionitis show a wide range of both aerobic and anaerobic bacteria [3, 4]. These include *Streptococcus pyogenes* and *Streptococcus agalactiae*, both well-known causes of maternal peripartum infection [5, 6]. In contrast, *S. dysgalactiae* is not as well-established as a cause of maternal peripartum infection but has been described in several studies. For example, a French report describing 182 cases of *S. dysgalactiae* infections identified three cases of endometritis [7]. Another study on infections caused by Group G streptococci reported four cases of puerperal sepsis [8]. A Norwegian study identified β-haemolytic streptococci in cultures over a 15-year period, where 282 of 4935 non-invasive group C or G streptococci cases were reported to have a “postpartum/genitalia focus” [9]. No detailed clinical data for these cases were reported, however. In addition, a study from Finland described an increased risk of postpartum endometritis if pregnant women were colonized with group C or G streptococci prior to delivery [10]. In that study, eighteen cases of group C or G streptococci endometritis were reported.

*S. dysgalactiae* can be divided into *emm-*types, depending on the sequence of the gene encoding the hypervariable NH_2_-terminus of the M protein [11]. Over 60 types have been identified, and the most common types vary by country [2]. In Sweden and Norway, *stG62647* has emerged as the predominant type in recent years [12, 13]. Some suspected outbreaks of *S. dysgalactiae* infections in healthcare facilities have been described. A British study identified 63 suspected outbreaks of group C or G infections over a period of 6 years in hospitals, outpatient clinics, schools and nursing homes, including 12 suspected outbreaks at maternity units [14]. A Danish case report details two cases of group G streptococcal infection with type *stG643* in two mothers who had shared a bathroom on the same delivery ward [15]. In this population-based, retrospective study, we aimed to investigate the incidence, clinical manifestation, and outcome of maternal peripartum infections caused by *S. dysgalactiae* in a healthcare region in the south of Sweden. Further, we aimed to investigate the distribution of *emm-*types in relation to severity of disease, and to use whole genome sequencing (WGS) to examine suspected hospital outbreaks.

## Materials and methods

### Microbiology and inclusion criteria

The clinical microbiology laboratory in Lund, Sweden, serves all ten hospitals in the Region Skåne, which had a population of approximately 1 389 000 in December 2020. Searches in the laboratory’s database identified patients with growth of Lancefield group C or G streptococci or *S. dysgalactiae* in a culture, collected between January 2014 and September 2020, at any of the region’s five departments for gynecology and obstetrics. For the years 2014–2016, species identification was based on colony morphology and a positive latex agglutination test for Lancefield group antigen C or G. For the years 2017–2020, MALDI-TOF MS was used as the primary species identification method [16]. As part of the laboratory routine, all isolates had previously been subjected to *emm-*typing. The patients identified in this search were assessed for eligibility. Only patients with culture results from the genital tract or from a caesarian section wound were included. Patients with culture results solely in a urine sample were thus not included. Patients with multiple positive cultures within a four-week period were regarded as having only one episode. Only patients who were either pregnant or had given birth or had undergone an abortion within 42 days prior to debut of clinical symptoms were included. Patients were excluded if another focus of infection was identified, either in the form of a positive blood culture with a different pathogen, or if clinical signs and symptoms of a concurrent infection outside of the genital tract were present. Patients with a polymicrobial vaginal or cervical cultures were not excluded except for those with concurrent growth of *S. pyogenes*.

### DNA extraction and sequencing

The bacterial isolates had been stored in a freezer at −80 °C. The isolates were collected from the freezer and cultivated overnight on blood agar plates at 35^0^ C. Approximately 3–4 colonies were collected with a plastic inoculation loop and DNA extraction was performed using the DNeasy Blood and tissue kit (Qiagen) according to instructions from the manufacturer. After the DNA extraction, the DNA concentration was measured with Qubit Broad range and High Sensitivity dsDNA Assay (Thermo Fisher Scientific) according to the manufacturer’s protocol. Fluorescence was measured using the Qubit fluorometer, and DNA concentrations were calculated based on a standard curve. Genomic libraries were prepared using the Illumina DNA Prep kit, following the manufacturer’s protocol (Illumina). Briefly, DNA was fragmented, and adapter ligation was performed to enable the binding of sequences required for amplification and sequencing. The libraries were then amplified and purified to ensure the appropriate fragment sizes. The quality and concentration of the libraries were assessed using a Qubit fluorometer for quantification and an Agilent TapeStation for fragment size distribution. Finally, the libraries were sequenced on an Illumina NextSeq 2000 platform using a P1 300-cycle flow cell, generating paired-end reads with a read length of 150 bp.

### Bioinformatic analysis

FastQC (version 0.11.9) was used for quality control of the reads and Kraken (version 2.1.3 with the standard database) was used to assign taxonomic labels to the reads. Genome assembly was performed with SPAdes (version 3.15.5) and Quast (version 5.3) was used for quality control. Mapping of FASTQ-files to the *S. dysgalactiae* reference genome (RefSeq GCF_016128095.1) was carried out with Burrows-Wheeler aligner (BWA, version 0.7.15) for quality analysis. Snippy (version 4.6.0) with default settings was used as a pipeline for mapping (reference genome RefSeq GCF_016128095.1) and variant calling. The phylogenetic tree was generated with FastTree (version 2.1.11), which is an approximate and fast method for constructing phylogenetic trees based on maximum likelihood principles.

### Review of medical records

Patient records were examined, and data were recorded regarding prior medical conditions, details of birth (including week of gestation, mode of delivery, blood loss, duration of labor after rupture of membranes, meconium stained amnionic fluid, laceration grade, use of episiotomy, instrument assisted delivery, or use of antibiotic prophylaxis), date of delivery or abortion, place of delivery, infection symptoms and date of debut, laboratory results, vital signs and antibiotic therapy. The outcomes were defined as length of hospital stay, intensive care unit (ICU) care, need of surgery or death.

### Definition of infection and sepsis

Endometritis was defined according to the CDC’s (Centers for Disease Control and Prevention) criteria with some modification: *S. dysgalactiae* cultured from the genital tract, and at least one of the following signs: abdominal pain, uterine tenderness or purulent discharge from uterus with no other recognized cause [17]. Postpartum fever was defined as fever without any localized signs or symptoms of infection. Findings of *S. dysgalactiae* without any symptoms as described above was defined as suspected colonization. Chorioamnionitis was defined as fever with a debut between rupture of membranes and delivery, with a diagnosis of chorioamnionitis made by the patient’s physician. Wound infections were identified according to physician’s clinical assessment and description of signs of infection. The Sepsis-3 criteria were used according to the definitions in Singer et al. [18].

### Cluster definition

A cluster was defined as two or more patients with puerperal infection caused by *S. dysgalactiae* of the same *emm-*type who had been cared for at the same hospital within a 30-day period. The isolates from these patients were subjected to whole genome sequencing and phylogenetic analysis, together with 12 *S. dysgalactiae* control isolates. A control isolate had to have the same *emm*-type as an isolate from a cluster, collected within the same period, but should have no apparent epidemiological connection with the cluster isolate.

### Statistical analysis

Data were analyzed using GraphPad Prism 9.4.0. Values are presented as frequencies or as median with interquartile ranges (IQR). Fischer’s exact test was used to compare groups.

## Results

### Study cohort

**Fig. 1:**
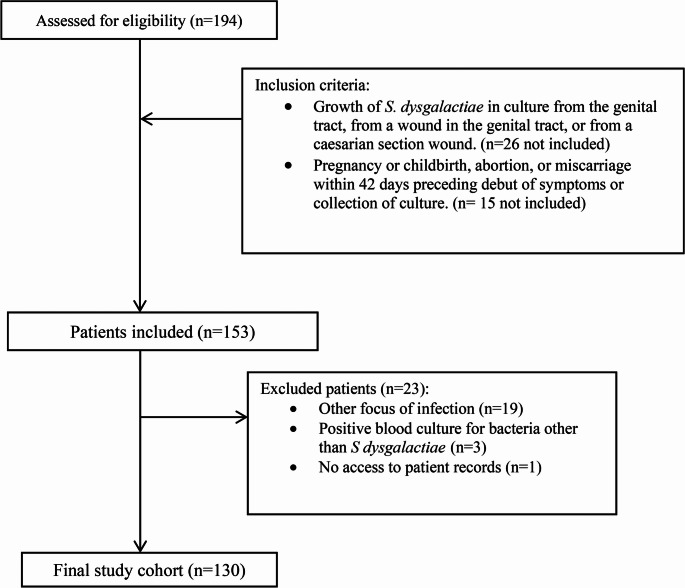
Study enrolment

Patient inclusion and exclusions are shown in Fig. 1. The final study cohort comprised 130 episodes in 130 patients. During the study period, 109 308 births took place in Region Skåne, averaging 16 412 births per year. The incidence of *S. dysgalactiae* postpartum infection would then correspond to approximately 1 case/1000 births.Background data and details of delivery including risk factors for infection are summarized in Table 1.

**Table 1:** Background data of the study population

Age (years)	31 (28–34)*
No. of births	2 (1–2)*
No. nulliparous	9 (8%)
Gestational diabetes	6 (5%)
Type 2 diabetes	1 (1%)
Number postpartum	114 (88%)
Number post-abortion	16 (12%)
spontaneous abortion	2 (12.5%)
medical abortion	14 (78.5%)
*Details of delivery*	
Caesarean section	10 (8%)
planned caesarean	2 (20%)
emergency caesarean	8 (80%)
Vaginal delivery	104 (91%)
Induction of labor	37 (33%)
Gestational age (weeks)	40 (39–41)*
No. of pre-term births (before week 37 + 0)	8 (7%)
Duration of labor after rupture of membranes^1^	5 h (1.6–11)*
Meconium-stained amniotic fluid^1^	34 (33%)
Use of vacuum extraction	10 (10%)
Blood loss^2^	400 ml (300–550)*
Perineal tear	
Grade 1	34 (33%)
Grade 2	47 (45%)
Grade 3	2 (2%)
Grade 4	0
Episiotomy	6 (6%)
Manual placenta removal	7 (7%)
Antibiotic prophylaxis during labor/C-section	18 (16%)

^*^Median and IQR

^1^Data missing in eleven cases

^2^Data missing in four cases

^3^Data missing in three cases

### Focus of infection and outcome

Table 2 shows a summary of the focus and severity of infection, outcome and treatment. A total of 94 patients fulfilled criteria for endometritis, eight patients had a wound infection, four patients were diagnosed with chorioamnionitis and fifteen patients developed postpartum fever without fulfilling the criteria for a localized infection. Eleven patients had a vaginal or cervical culture with growth of *S. dysgalactiae* but did not have fever or other signs of infection and were categorized as being colonized with *S. dysgalactiae*.

**Table 2:** Focus of infection and outcome

Type of infection^1^	
Endometritis	94 (72%)
Chorioamnionitis	4 (2%)
Postpartum fever	15 (12%)
Wound infection	8 (6%)
*Suspected colonization*	11 (8%)
*Details of infection*^2^	
Days from event to debut of symptoms^3, 4^	4 (3–6) days^5^
Early debut endometritis (< 48 h)	12 (13%)
Late debut endometritis (> 48 h)	82 (87%)
Initial CRP (mg/L)^6^	63 (29–96)^5^
Maximum CRP (mg/L)^7^	141 (91–197)^5^
Average white blood cell count (10^9^/L)^8^	14 (11.9–16.5)^5^
Sepsis (according to Sepsis-3)	1 (1%)
*S. dysgalactiae* bacteremia	5 (4%)
*Outcome*	
Hospital admission^4^	71 (55%)
Admission to intensive care unit	0
Death	0
Surgical intervention	7
Intravenous antibiotic treatment^1^	
Number of patients	65 (55%)
Length of treatment	3 (2–3) days^5^
Per oral antibiotic treatment^1^	
Number of patients	114 (96%)
Length of treatment	10 (7–10) days^5^
Total days antibiotics	10 (10–11) days^5^
*Microbiology*	
Positive blood culture	5 (4%)
Monomicrobial culture	109 (84%)
Polymicrobial culture	21 (16%)

^1^Including two patients diagnosed with simultaneous endometritis and wound infection

^2^Patients with suspected colonization are excluded

^3^Patients with chorioamnionitis are excluded

^4^Data missing in 1 case

^5^Median and IQR

^6^Data missing in 7 cases

^7^Data missing in 37 cases

^8^Data missing in 39 cases

In total, 65 patients (55%) were admitted to or remained in hospital due to the infection. No patients required admission to the ICU. Seven patients required some form of surgical procedure, typically dilation and curettage or suction curettage.

### Microbiology and emm-types

Susceptibility to erythromycin and clindamycin was recorded. In total 2 of 32 (6%) and 2 of 56 (4%) of tested isolates showed resistance to erythromycin and clindamycin, respectively. *S. dysgalactiae* was the only reported microorganism in 109 cultures (84%) (Table 2). In addition to *S. dysgalactiae*, 21 cultures had findings of other bacteria such as *Escherichia coli* and other *Enterobacterales* species, *Staphylococcus aureus* or group B streptococci. Seven of the polymicrobial cultures were from patients categorized as colonized with *S. dysgalactiae* without clinical symptoms of infection.

The most common *emm-*types were *stG62647* (*n* = 41), followed by *stG485* (*n* = 15) and *stC74A* (*n* = 14). Table 3 summarizes focus of infection and outcome in relation to *emm*-types.

**Table 3:** Focus of infection and outcome in relation to *emm*-types

emm-type	All types	stG62647	stG485	stC74A	stG643	stG6	stG2078	stG480	Other^1^
Total number, *n* = 130	*n* = 130	*n* = 41	*n* = 15	*n* = 14	*n* = 13	*n* = 11	*n* = 10	*n* = 10	*n* = 16
Type of infection^2, 3^:									
- Endometritis	94 (72%)	28 (68%)	9 (60%)	11 (79%)	10 (78%)	10 (91%)	9 (90%)	7 (70%)	10 (63%)
- Chorioamnionitis	4 (3%)	2 (5%)	0	1 (7%)	0	0	0	0	1 (6%)
- Postpartum fever	15 (12%)	4 (10%)	3 (20%)	0	1 (8%)	0	1 (10%)	2 (20%)	4 (25%)
- Wound infection	8 (6%)	3 (7%)	2 (13%)	1 (7%)	0	1 (9%)	0	1 (10%)	0
- Suspected colonization	11 (8%)	5 (12%)	2 (13%)	1 (7%)	2 (15%)	0	0	0	1 (6%)
Early debut endometritis (< 48 h)	12 (9%)	4 (14%)^4^	1 (11%)	0	0	1 (10%)	4 (44%)^5^	2 (29%)	0
Late debut endometritis (> 48 h)	82 (63%)	24 (86%)^4^	8 (89%)	11 (100%)	10 (100%)	9 (90%)	5 (56%)	5 (71%)	10 (100%)
*S. dysgalactiae* bacteremia	5 (4%)	3 (7%)	1 (7%)	0	0	0	1 (10%)	0	0
Hospital admission^6^	71 (55%)	21 (51%)	9 (60%)	7 (50%)	5 (38%)	10 (91%)^7^	8 (80%)	6 (60%)	5 (31%)

^1^*emm*-types with fewer than 10 cases are grouped as Other. *emm*-types are *stG222* (*n* = 1), *stC839* (*n* = 1), *stG10* (*n* = 3), *stG11* (*n* = 3), *stG245* (*n* = 1), *stG652* (*n* = 4), *stG6792* (*n* = 1) and *stG840* (*n* = 2)

^2^Including two patients diagnosed with simultaneous endometritis and wound infection

^3^Stastistical significance of *emm*-type in relation to type of infection not analyzed

^4^Expressed as percentages of cases of endometritis

^5^*stG2078* more common in early debut endometritis with statistical significance (*p* = 0.01) using Fisher’s exact test

^6^Data missing in 1 case

^7^*stG6* more common in hospitalised patients with statistical significance (*p* = 0.02) using Fisher’s exact test

### Cluster analysis results

**Fig. 2:**
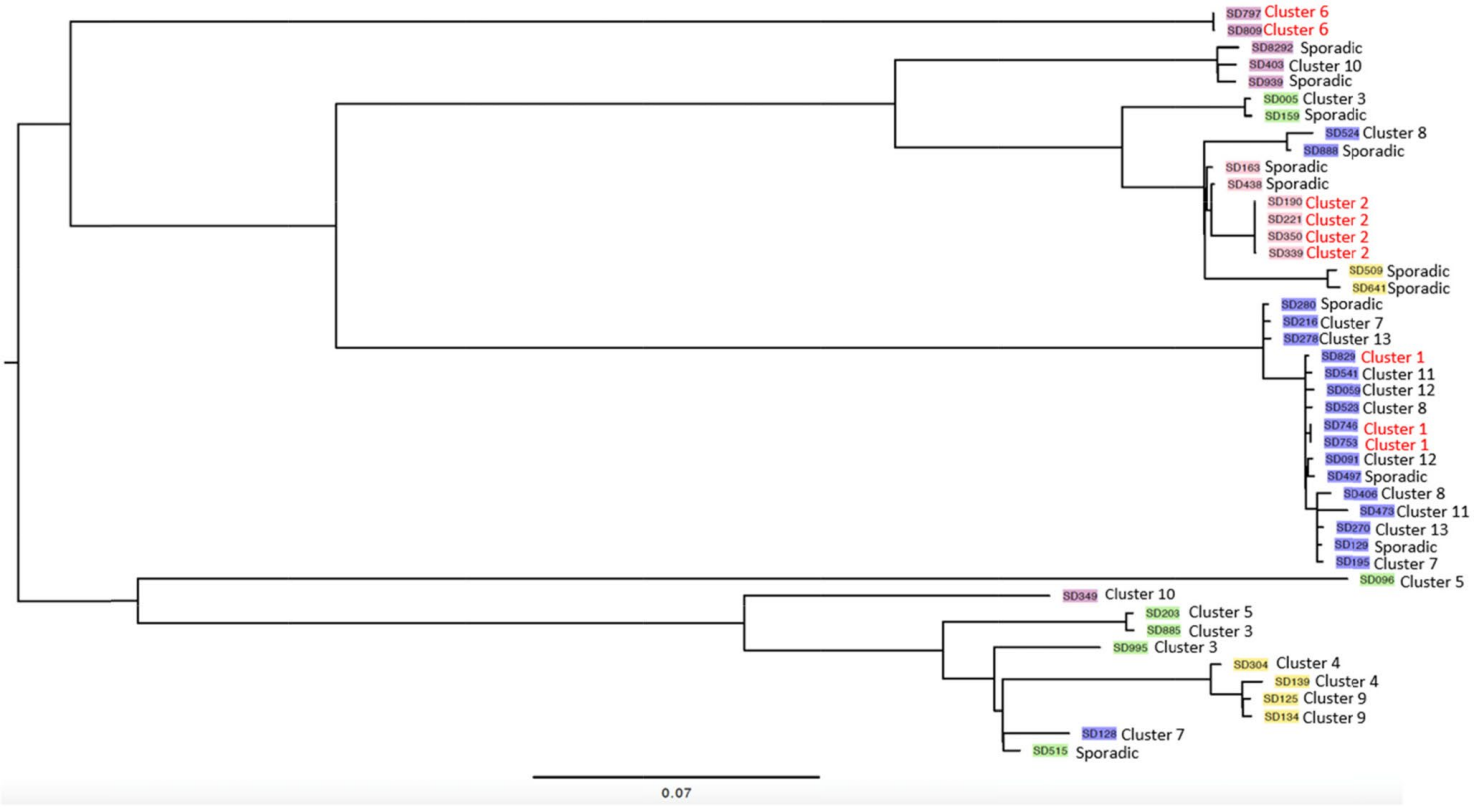
Phylogenetic tree with isolates from suspected hospital clusters

The identified clusters are detailed in Table 4. According to our definition, 13 clusters representing suspected outbreaks were identified, with two to four patients in each cluster. The proportion of patients with a given *emm*-type associated with clusters was determined and compared. For patients infected with *stG62647*, a higher proportion (37%) was associated with clusters as compared to patients infected with other *emm*-types (19%) (*p* = 0.048). Corresponding comparisons for the other *emm*-types did not yield any significant differences. The phylogenetic analysis showed that despite that the clusters contained isolates with the same *emm-*type, only three of the thirteen clusters (cluster 1, 2 and 6) contained isolates that were probably closely genetically related (0–6 single nucleotide polymorphisms (SNP) differences). In Fig. 2, the phylogenetic tree shows the relationship between all isolates from clusters and the twelve control isolates. The different genomes are colored according to *emm*-type. As can be seen, not all isolates of the same *emm-*type were clustered within this tree. For instance, most *stG62647* isolates (blue) are found in a major central cluster, but there are three *stG62647* isolates placed elsewhere in the tree.

**Table 4:** Description of thirteen *S. dysgalactiae* hospital clusters

Cluster	Year	Patient (pt)	Case description	culture locations	emm type	SNPs distance to other isolates in cluster
1	2014	1	Vaginal delivery at hospital B	Cervix, urine	stG62647	2, 58
1	2014	2	Vaginal delivery at hospital B on the same day	Cervix, urine	stG62647	2, 58
1	2014	3	Vaginal delivery at hospital B 26 days after pt. 1 and 2	Cervix	stG62647	58
2	2017	4	Vaginal delivery at hospital B	Cervix, urine	stG2078	0, 6
2	2017	5	Vaginal delivery at hospital B three days after pt. 4	Cervix, urine	stG2078	0, 6
2	2017	6	Vaginal delivery at hospital B 29 days after pt. 5	Cervix, urine	stG2078	6
2	2017	7	Complicated vaginal delivery at hospital B four days after pt. 6	Cervix, urine	stG2078	0, 6
3	2017	8	Vaginal delivery at hospital C	Cervix, urine	stG485	2294, 9049
3	2017	9	Vaginal delivery at hospital C 27 days after pt. 8	Vagina	stG485	2294, 9285
3	2017	10	Vaginal delivery at hospital C 3 days after pt. 9	Vagina	stG485	9049, 9285
4	2018	11	Complicated vaginal delivery at hospital A	Vagina	stC74A	407
4	2018	12	Medical abortion at hospital A outpatient clinic 30 days after pt. 11	Cervix	stC74A	407
5	2018	13	Complicated vaginal delivery at hospital C	Vagina	stG485	10,495
5	2018	14	Vaginal delivery at hospital C 21 days after pt. 13	Cervix	stG485	10,495
6	2018	15	Vaginal delivery at hospital B	Cervix, urine	stG6	6
6	2018	16	Vaginal delivery at hospital B 2 days after pt. 15	Cervix	stG6	6
7	2018	17	Medical abortion at hospital A	Vagina	stG62647	11,495, 11,670
7	2018	18	Vaginal delivery at hospital A 7 days after pt 17	Cervix, urine	stG62647	441, 11,670
7	2018	19	Medical abortion at hospital A 19 days after pt. 18	Cervix	stG62647	441, 11,495
8	2018	20	Vaginal delivery at hospital C	Cervix, urine	stG62647	213, 9208
8	2018	21	Vaginal delivery at hospital C 27 days after pt. 20	Cervix	stG62647	213, 9194
8	2018	22	Vaginal delivery at hospital C 10 days after pt. 21	Vagina, urine	stG62647	9194, 9208
9	2019	23	Medical abortion at hospital A outpatient clinic	Cervix	stC74A	109
9	2019	24	Pre-term birth at hospital A two days after pt. 23	Cervix, urine	stC74A	109
10	2019	25	Vaginal delivery at hospital A	Cervix	stG6	9316
10	2019	26	Vaginal delivery at hospital A 8 days after pt. 25	Cervix	stG6	9316
11	2019	27	Vaginal delivery at hospital D	Blood, cervix, urine	stG62647	322
11	2019	28	Vaginal delivery converted to emergency caesarean section at hospital D 14 days after pt. 27	Uterus	stG62647	322
12	2019	29	Vaginal delivery in hospital E	Vagina	stG62647	101
12	2019	30	Vaginal delivery in hospital E 10 days after pt. 29	Wound culture	stG62647	101
13	2019	31	Complicated vaginal delivery in hospital A	Cervix	stG62647	457
13	2019	32	Vaginal delivery in hospital A eight days after pt 31	Cervix	stG62647	457

## Discussion

This is the first large-scale study on *S. dysgalactiae* peripartum infections, describing clinical features and severity of infection in 130 patients. Our findings demonstrate that *S. dysgalactaie* peripartum infections impact a relatively large number of patients and even though no patient was critically ill, the morbidity appeared to be substantial.

The incidence of *S. dysgalactiae* postpartum infections in our study was approximately 1 case per 1000 births. In the study by Jaamala et al., where 24 cases of *S. dysgalactiae* postpartum infections were reported, the prevalence of *S. dysgalactiae* colonization in pregnant women was 2.9%, but the incidence of *S. dysgalactiae* postpartum infections was not reported in the study [10]. However, based on numbers presented, the incidence in the report by Jaamala et al. would correspond to approximately 2.3 cases per 1000 births, which is higher than what we report in this study. This might represent a true difference in incidence between different health care settings but could also be due to differences in study design and inclusion criteria. For instance, it is possible that patients with mild symptoms contacted other healthcare facilities and therefore were missed in our study. A prospective study design is most likely required to determine the true incidence of *S. dysgalactiae* postpartum infections.

The clinical features of *S. dysgalactiae* postpartum infections have not been well described in most previous studies [7, 9]. Our study suggests that endometritis is the most common clinical presentation (*n* = 94, 72%), and most of these patients presented with a late onset of symptoms (*n* = 82, 87%). The average time from delivery to symptoms for all patients was 5.2 days which is in line with what was reported in Jaamala et al. [10].

In Sweden, the first-line treatment for systemic infection when endometritis is suspected is a third-generation cephalosporin in combination with metronidazole. Patients with endometritis who do not require hospitalization may be treated orally with amoxicillin/clavulanic acid and metronidazole. *S. dysgalactiae* is universally sensitive to penicillins but sensitivity against macrolides, which is a common treatment option for penicillin-allergic patients, vary between countries. In our study, only a few percent of the tested isolates were resistant against erythromycin (6%) or clindamycin (4%), which differs from what was reported for example in a French study, where the rate of erythromycin resistance was 26% [7].

As in previous research on *S. dysgalactiae emm*-types in Sweden and Norway [12, 13], the most common *emm*-type encountered was *stG62647*, accounting for 32% of isolates overall and three of five blood culture isolates. In Norway, *stG62647* has been associated with more severe disease compared to other *emm*-types. Interestingly, we did not find a significant difference in frequency of *stG62647* isolates when comparing the distribution of *emm*-types in hospitalized vs. non-hospitalized patients, or in patients with early vs. late onset of endometritis symptoms. Instead, *stG6* was overrepresented among patients requiring hospitalization and *stG2078* among patients with an early onset of endometritis. Despite that our study is comparatively large, we cannot exclude that true associations could be missed due to lack of power and that false associations are found due to multiple comparisons.

Our initial findings of clustered cases of the same *emm-*type made us suspect that *S. dysgalactiae* peripartum infection outbreaks could be common. However, the detailed analysis with WGS showed that most isolates were not clonally related, refuting clonal outbreaks in all but three clusters. This demonstrates the value of WGS and shows that true outbreaks with a likely source of spread within the hospitals were uncommon. The fact that isolates of the same *emm-*type did not always display a close genetic relationship in our phylogenetic analysis, is most likely explained by events of homologous recombination of the *emm*-gene as has been suggested previously [19]. Hence, a collection of *S. dysgalactiae* isolates from the same geographic area, with an identical *emm*-type, may in fact constitute a diverse genetic population of clones, possibly with different virulence factors.

To our knowledge, this is the largest study on *S. dysgalactiae* peripartum infections. The patients were identified in a healthcare-region served by one clinical microbiology laboratory during the whole study period, which in combination with population size data allow for calculation of incidence. Also, all isolates had been *emm*-typed and stored, which made it possible to investigate the suspected clusters with WGS and phylogenetic analysis. This study has some limitations, of which most are related to the retrospective study design. Some patients with *S. dysgalactiae* peripartum infections might have been cultured at other healthcare facilities than the ones included in our search. There could also have been cases that sought care at the included healthcare facilities but were not sampled from the genital tract. For instance, patients with endometritis and growth of *S. dysgalactiae* only in blood culture were not included in our study. This could have led to an underestimation of incidence. Also, there were missing data in medical records and in one case failure to gain access to the medical record.

Due to our study design, we did not have a control group for comparison of different outcomes. For instance, we could not compare the clinical presentation of endometritis caused by *S. dysgalactiae* with endometritis caused by other bacterial species, such as GAS, or estimate the risk for adverse pregnancy outcomes when colonized or infected by *S. dysgalactiae*. To answer these questions, further studies are required.

To conclude, *S. dysgalactiae* peripartum infections are associated with substantial morbidity but seems to have a favorable outcome when diagnosed and treated promptly with antibiotics. Hospital outbreaks of *S. dysgalactiae* peripartum infections are rare, but when suspected, WGS should be employed to confirm or dismiss relatedness between isolates.

## Data Availability

The data that support the findings of this study are available from the corresponding author upon reasonable request.

## References

[1] Brandt CM, Spellerberg B (2009) Human infections due to *Streptococcus dysgalactiae* subspecies equisimilis. Clin Infect Dis 49(5):766–772 19635028 10.1086/605085

[2] Rantala S (2014) *Streptococcus dysgalactiae subsp. Equisimilis* bacteremia: an emerging infection. Eur J Clin Microbiol Infect Dis 33(8):1303–1310 24682845 10.1007/s10096-014-2092-0

[3] Kim CJ, Romero R, Chaemsaithong P, Chaiyasit N, Yoon BH, Kim YM (2015) Acute chorioamnionitis and funisitis: definition, pathologic features, and clinical significance. Am J Obstet Gynecol 213(4 Suppl):S29-52 26428501 10.1016/j.ajog.2015.08.040 PMC4774647

[4] Mackeen AD, Packard RE, Ota E, Speer L (2015) Antibiotic regimens for postpartum endometritis. Cochrane Database Syst Rev 2015(2):Cd001067 25922861 10.1002/14651858.CD001067.pub3 PMC7050613

[5] Muller AE, Oostvogel PM, Steegers EA, Dörr PJ (2006) Morbidity related to maternal group B streptococcal infections. Acta Obstet Gynecol Scand 85(9):1027–1037 16929406 10.1080/00016340600780508

[6] Rottenstreich A, Benenson S, Levin G, Kleinstern G, Moses AE, Amit S (2019) Risk factors, clinical course and outcomes of pregnancy-related group A Streptococcal infections: retrospective 13-year cohort study. Clin Microbiol Infect 25(2):251. .e1-.e4 10.1016/j.cmi.2018.10.002 30336220

[7] Loubinoux J, Plainvert C, Collobert G, Touak G, Bouvet A, Poyart C (2013) Adult invasive and noninvasive infections due to *Streptococcus dysgalactiae subsp. Equisimilis* in France from 2006 to 2010. J Clin Microbiol 51(8):2724–2727 23698531 10.1128/JCM.01262-13 PMC3719644

[8] Vartian C, Lerner PI, Shlaes DM, Gopalakrishna KV (1985) Infections due to Lancefield group G streptococci. Med Baltim 64(2):75–88 10.1097/00005792-198503000-00001 3974442

[9] Oppegaard O, Mylvaganam H, Kittang BR (2015) Beta-haemolytic group A, C and G streptococcal infections in Western Norway: a 15-year retrospective survey. Clin Microbiol Infect 21(2):171–178 25658557 10.1016/j.cmi.2014.08.019

[10] Jaalama M, Palomäki O, Vuento R, Jokinen A, Uotila J (2018) Prevalence and clinical significance of Streptococcus dysgalactiae subspecies equisimilis (Groups C or G Streptococci) colonization in pregnant women: A retrospective cohort study. Infect Dis Obstet Gynecol 2018:2321046 29973773 10.1155/2018/2321046 PMC6008822

[11] Centers for Disease Control and Prevention *Streptococcus* laboratory (www.cdc.gov/strep-lab)

[12] Oppegaard O, Mylvaganam H, Skrede S, Lindemann PC, Kittang BR (2017) Emergence of a *Streptococcus dysgalactiae subspecies equisimilis* stG62647-lineage associated with severe clinical manifestations. Sci Rep 7(1):7589 28790435 10.1038/s41598-017-08162-z PMC5548910

[13] Trell K, Nilson B, Rasmussen M (2016) Species and *emm*-type distribution of group C and G Streptococci from different sites of isolation. Diagn Microbiol Infect Dis 86(4):467–469 27712926 10.1016/j.diagmicrobio.2016.09.008

[14] Efstratiou A (1989) Outbreaks of human infection caused by pyogenic streptococci of Lancefield groups C and G. J Med Microbiol 29(3):207–219 2746629 10.1099/00222615-29-3-207

[15] Jöhnk ML, Ingels HA, Sørensen AL, Lambertsen L (2013) [Gruppe G Streptococci as a rare cause of nosocomial post-partum infection]. Ugeskr Laeger 175(11):740–741 23480889

[16] Cherkaoui A, Emonet S, Fernandez J, Schorderet D, Schrenzel J (2011) Evaluation of matrix-assisted laser desorption ionization-time of flight mass spectrometry for rapid identification of Beta-hemolytic Streptococci. J Clin Microbiol 49(8):3004–3005 21697322 10.1128/JCM.00240-11 PMC3147758

[17] Horan TC, Andrus M, Dudeck MA (2008) CDC/NHSN surveillance definition of health care-associated infection and criteria for specific types of infections in the acute care setting. Am J Infect Control 36(5):309–332 18538699 10.1016/j.ajic.2008.03.002

[18] Singer M, Deutschman CS, Seymour CW, Shanker-Hari M, Annane D, Bauer M et al (2016) The third international consensus definitions for sepsis and septic shock (Sepsis-3). JAMA 315(8):801–810 26903338 10.1001/jama.2016.0287 PMC4968574

[19] Senneby E, Hallström B, Rasmussen M (2021) Genetic relatedness of *Streptococcus dysgalactiae* isolates causing recurrent bacteraemia. J Med Microbiol 70(3). 10.1099/jmm.0.001330 10.1099/jmm.0.001330 33616518

